# Common variants of vitamin D receptor gene polymorphisms and risk of gastric cancer: A meta-analysis

**DOI:** 10.1097/MD.0000000000039527

**Published:** 2024-08-30

**Authors:** Min Guan, Yong Wang

**Affiliations:** a Department of Gastrointestinal Surgery, Shandong Provincial Third Hospital Affiliated to Shandong University, Jinan, Shandong, China; b Department of Hepatobiliary Surgery, Shandong Provincial Third Hospital Affiliated to Shandong University, Jinan, Shandong, China.

**Keywords:** gastric cancer, meta-analysis, polymorphisms, vitamin D receptor

## Abstract

**Background:**

While earlier studies have suggested that variations in the vitamin D receptor (VDR) gene could influence the susceptibility to gastric cancer (GC), the results have shown inconsistency. This meta-analysis aimed to examine the association of 5 common polymorphisms in VDR, including Taq1 rs731236 (T > C), FokI rs2228570 (C > T), Cdx2 rs11568820 (G > A), BsmI rs1544410 (G > A), and ApaI rs7975232 (G > T) with the risk of GC.

**Methods:**

A comprehensive search was carried out in PubMed, Web of Science, and Scopus to identify relevant studies published until January 2024. Odds ratios (ORs) with 95% confidence intervals (CIs) were utilized to assess the magnitude of associations.

**Results:**

Nine studies, with 2837 participants (1215 GC cases and 1622 healthy controls), were eligible. The FokI rs2228570 polymorphism showed a significant correlation with heightened susceptibility to GC under the recessive model (OR = 1.52; 95% CI: 1.06–2.19) and homozygote comparison (TT vs CC; OR = 1.59; 95% CI: 1.09–2.31). Taq1 rs731236 was also linked to an elevated risk of GC under the same models (recessive OR = 1.65; 95% CI: 1.14–2.39; homozygote OR = 1.68; 95% CI: 1.11–2.54). In the sensitivity analysis, when studies not adhering to Hardy–Weinberg equilibrium were excluded, the relationship between FokI rs2228570 polymorphism and GC disappeared, while the association for Taq1 rs731236 remained consistent. No significant association was identified for BsmI rs1544410, ApaI rs7975232, and Cdx2 rs11568820.

**Conclusion:**

This study revealed that FokI rs2228570 and Taq1 rs731236 polymorphisms of VDR might be linked to the odds of GC.

## 1. Introduction

Gastric cancer (GC), ranking as the fourth most prevalent cancer globally, continues to pose a substantial health issue, with high morbidity and mortality rates.^[1]^ Based on the GLOBOCAN 2020 evaluations, GC resulted in around 800,000 fatalities, representing 7.7% of total cancer-related deaths.^[2]^ The etiology of GC is multifactorial, involving complex interactions between genetic and environmental factors.^[3]^ The primary risk factor for GC is chronic infection with *Helicobacter pylori*.^[4]^ Alcohol consumption, being male, infection with Epstein Barr virus, increasing age, obesity, unhealthy diet, tobacco use, previous stomach surgery, and pernicious anemia have been involved in the ethology of GC.^[3,5–7]^ Furthermore, it is widely acknowledged that genetic factors may contribute to the development of GC.^[8]^

It has been indicated that a higher vitamin D level is linked to a lower risk of GC and it is believed to play a crucial role in suppressing the growth of g GC cells.^[9]^ Research suggests that GC patients often exhibit vitamin D deficiency.^[10]^ Vitamin D has the potential to impact cancer progression by affecting multiple signaling pathways related to cell apoptosis, metastasis, invasion, and proliferation.^[11]^ The function of vitamin D is mediated through the vitamin D receptor (VDR). The VDR is situated on the chromosome region 12q13.1, spanning about 75 kilobases with a length of 63,493 nucleotides.^[12]^ While multiple genetic single nucleotide polymorphisms (SNP) have been discovered in the VDR gene, the FokI (rs2228570 C > T), TaqI (rs731236 T > C), BsmI (rs544410 G > A), ApaI (rs7975232 G > T), and Cdx2 (rs11568820 G > A) are the most prevalent variations, and could alter the ability of vitamin D to bind to the receptor by impacting VDR expression. Accordingly, their correlation with the risk of cancer have been studied broadly. VDR genetic variations have been recognized to be related to the susceptibility to different types of malignancies like colon, breast, lung, and ovarian.^[13–16]^ However, the influence of VDR gene changes on the susceptibility to GC remains uncertain. Some studies have explored the correlation between VDR gene SNPs and the susceptibility to GC, yet the findings have shown variability with positive^[17]^ and null^[7]^ associations. The discrepancies in the results of prior studies could be attributed to variations in sample size, geographic location, and ethnicity. This meta-analysis sought to quantitatively assess the connection between VDR gene polymorphisms and the susceptibility to GC.

## 2. Materials and methods

study was carried out by following the PRISMA guidelines.^[18]^ This review used the data from the previously published studies and ethical approval was not applicable for this type of study.

### 2.1. Search strategy

A thorough search without language restrictions was carried out in PubMed, Web of Science, and Scopus to identify pertinent studies published up to January 2024. with the use of the following search strategy: ((((((“Receptors, Calcitriol”[Mesh]) OR (Calcitriol Receptor[Title/Abstract])) OR (vitamin d receptor[Title/Abstract])) OR (VDR[Title/Abstract])) AND (((((((((“Polymorphism, Genetic”[Mesh] OR “Polymorphism, Single Nucleotide”[Mesh]) OR (Polymorphism*[Title/Abstract])) OR (variation*[Title/Abstract])) OR (variant*[Title/Abstract])) OR (mutation[Title/Abstract])) OR (SNP[Title/Abstract])) OR (SNPs[Title/Abstract])) OR (allele[Title/Abstract])) OR (genotype[Title/Abstract]))) AND ((stomach[Title/Abstract]) OR (gastric[Title/Abstract]))) AND ((((((tumor[Title/Abstract]) OR (cancer[Title/Abstract])) OR (neoplasm[Title/Abstract])) OR (neoplasia[Title/Abstract])) OR (carcinoma[Title/Abstract])) OR (adenocarcinoma[Title/Abstract])). Moreover, the citations in pertinent articles were manually reviewed to discover any additional suitable studies.

### 2.2. Inclusion and exclusion criteria

The studies were considered eligible based on the following criteria: assessed the association of VDR SNPs with the odds of GC, studies were case–control in design, reported genotype frequencies of SNPs in both cases and controls, and studies were conducted on human subjects. Experiments on animals, reviews, comments, letters, republished studies, editorials, and studies with unextractable data on genotype frequencies in cases and controls were excluded. Two independent researchers conducted the study selection process and arrived at a final consensus in case of disagreement through discussion.

### 2.3. Data extraction and quality assessment

Data collected from each study included the first author’s name, the Hardy–Weinberg equilibrium (HWE) status, genotype distribution for both cases and controls, total sample size, number of GC cases and healthy controls, country, ethnicity, percentage of males, and mean age of cases and controls. In cases where multiple studies on the same population were published sequentially, only the latest study was taken into consideration. The Newcastle-Ottawa Scale (NOS) was utilized to assess the methodological quality of the included studies employed to evaluate the quality of the publications included in the analysis.^[19]^ Studies with scores 4 to 6 were categorized as moderate quality and studies with scores 7 to 9 were classified as high quality based on their NOS score. The eligibility assessment and quality assessment processes were conducted by 2 authors independently, and any differences were fixed using a discussion.

### 2.4. Statistical analysis

To evaluate the adherence to the HWE in control groups, the chi-squared test was applied, where a *P*-value < .05 indicated significant disequilibrium. The risk of GC linked to the 5 common SNPs of the VDR gene was determined by pooling odds ratio (OR) along with its 95% confidence interval (CI) in 5 genetic models, comprising the dominant, allelic, heterozygote, recessive, and homozygote models. The heterogeneity was tested using the χ^2^ test, and a significance level of *P* < .10 was deemed significant. Due to the expected heterogeneity, the data was consolidated utilizing the random effects model. To gauge the dependability of the pooled effect sizes, a sensitivity analysis was carried out by excluding studies that did not adhere to the HWE from the primary analyses. To evaluate publication bias, funnel plots and Egger test were employed, with *P* values below .05 regarded as statistically significant. All analyses were performed using MetaGenyo web tool.^[20]^

## 3. Results

### 3.1. Study characteristics

**Figure 1.**
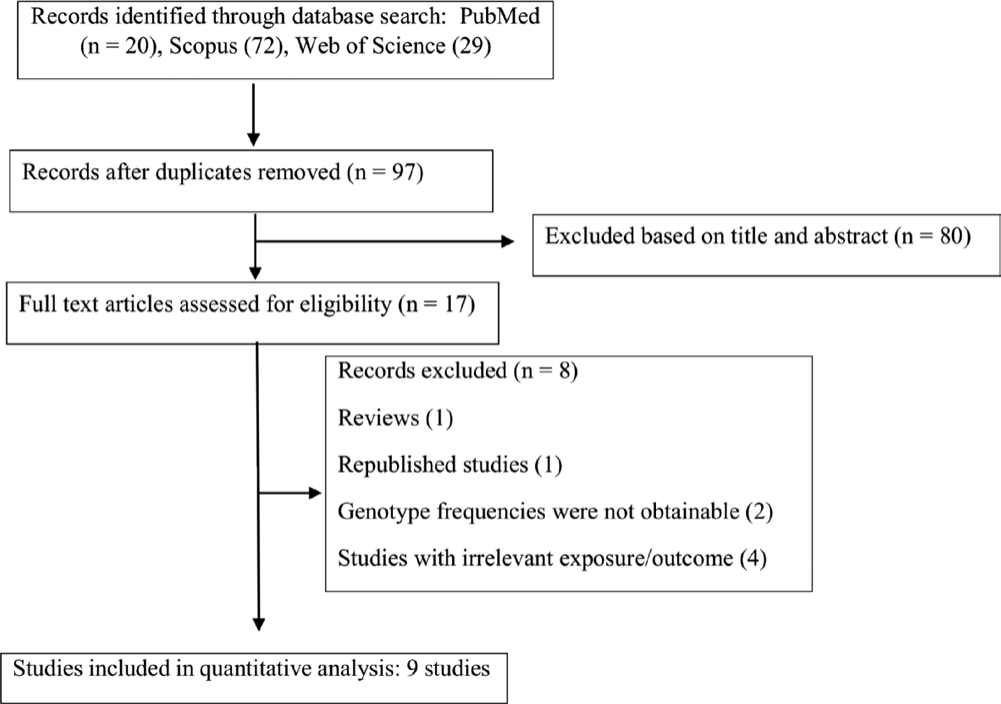
Flow diagram of the study.

The databases systematic search yielded 121 publications. Following the removal of 24 duplicates, an additional 80 studies were excluded due to irrelevant titles and abstracts. After a comprehensive full-text screening, 8 papers were excluded due to being irrelevant in terms of exposure or outcome, reviews, republished studies, or the genotype frequencies were not obtainable. Finally, 9 studies,^[7,12,13,17,21–25]^ with 2837 subjects (GC cases: 1215, healthy controls: 1622), exploring the associations of 5 common SNPs in VDR with GC were analyzed. The flow diagram of the screening process of evaluated publications is presented in Figure 1. Data for FokI rs2228570 (C > T) were reported in 7 studies,^[7,12,13,17,21–23]^ for Taq1 rs731236 (T > C) in 4 studies,^[7,12,23,24]^ for BsmI rs1544410 (G > A) in 2 studies,^[12,24]^ for ApaI rs7975232 (G > T) in 2 studies,^[12,24]^ and for Cdx2 rs11568820 (G > A) in 3 studies.^[13,22,25]^ The publication dates spanned from 2015 to 2021. The sample size of the included articles varied from 87 to 938 subjects. The mean age of cases was between 53.5 ± 7.92 and 65.06 ± 8.37 years. Most studies focused on Asian populations,^[12,13,17,21–25]^ with only 1 study examining Caucasians.^[7]^ The percentage of males in the studies populations ranged from 46.9% to 67.4%. In the controls of 5 studies, genotype distributions adhered to HWE,^[7,13,17,22,25]^ while in 4 studies, they exhibited deviations from HWE.^[12,21,23,24]^ According to the NOS scale, the quality of the analyzed studies was classified as moderate to high, with scores ranging from 5 to 9. The detailed information of the eligible publications is outlined in Table 1.

**Table 1:** Characteristics of eligible studies considered for the association between vitamin D receptor polymorphisms and GC risk in the meta-analysis.

Authors	Year	Country	Race	Population characteristics					Genotype frequency						P-HWE
				% Males	Cases	Controls	Case age	Control age	GC cases			Controls			
**FokI rs2228570 (C > T**)									**TT**	**TC**	**CC**	**TT**	**TC**	**CC**	
Durak	2019	Turkey	Caucasian	50.9	77	84	56.7 ± 1.74	51.42 ± 2.41	5	35	37	11	31	42	0.32
Parsamanesh	2018	Iran	Asian	56.8	69	100	57.8 ± 6.3	45.6 ± 4.3	5	18	80	3	343	110	0.001
Hoseinkhani	2021	Iran	Asian	51.7	99	100	56 ± 8.43	37 ± 7.43	43	24	32	24	36	40	0.02
Qadir	2021	India	Asian	60.7	143	150	55.3 ± 11.7	53.8 ± 11.8	27	72	44	18	62	70	0.54
Fang	2016	China	Asian	46.9	147	151	60.93 ± 10.96	60.12 ± 10.19	58	54	35	44	55	52	0.001
Yin	2017	China	Asian	67.4	330	608	65.06 ± 8.37	64.19 (±6.66)	66	153	97	115	299	166	0.49
Cong	2015	China	Asian	61.4	187	212	NR	NR	48	97	42	41	103	68	0.86
**Taq1 rs731236 (T > C**)									**CC**	**CT**	**TT**	**CC**	**CT**	**TT**	
Durak	2019	Turkey	Caucasian	50.9	77	84	56.7 ± 1.74	51.42 ± 2.41	19	38	20	14	40	30	0.91
Parsamanesh	2018	Iran	Asian	56.8	69	100	57.8 ± 6.3	45.6 ± 4.3	6	24	73	4	63	80	0.08
Qadir	2021	India	Asian	60.7	143	150	53.5 ± 7.92	51.2 ± 8.25	19	65	59	14	59	77	0.78
Hoseinkhani	2021	Iran	Asian	51.7	99	100	56 ± 8.43	37 ± 7.43	51	21	27	39	31	30	0.001
**BsmI rs1544410 (G > A**)									**AA**	**AG**	**GG**	**AA**	**AG**	**GG**	
Qadir	2021	India	Asian	60.7	143	150	53.5 ± 7.92	51.2 ± 8.25	38	64	41	32	54	64	0.002
Hoseinkhani	2021	Iran	Asian	51.7	99	100	56 ± 8.43	37 ± 7.43	17	20	62	20	29	51	0.006
**ApaI rs7975232 (G > T**)									**TT**	**TG**	**GG**	**TT**	**TG**	**GG**	
Qadir	2021	India	Asian	60.7	143	150	53.5 ± 7.92	51.2 ± 8.25	29	69	45	26	66	58	0.33
Hoseinkhani	2021	Iran	Asian	51.7	99	100	56 ± 8.43	37 ± 7.43	34	21	44	32	28	40	0.001
**Cdx2 rs11568820 (G > A**)									**AA**	**AG**	**GG**	**AA**	**AG**	**GG**	
Qadir	2021	India	Asian	60.7	143	150	55.3 ± 11.7	53.8 ± 11.8	9	55	79	6	66	76	0.21
Rehman	2021	Pakistan	Asian	NR	20	67	NR	NR	0	4	16	5	34	28	0.32
Yin	2017	China	Asian	67.4	330	608	65.06 ± 8.37	64.19 ± 6.66	69	162	99	114	283	193	0.57

P-HWE, *P*-value for Hardy–Weinberg equilibrium.

### 3.2. Quantitative data synthesis

**Figure 2.**
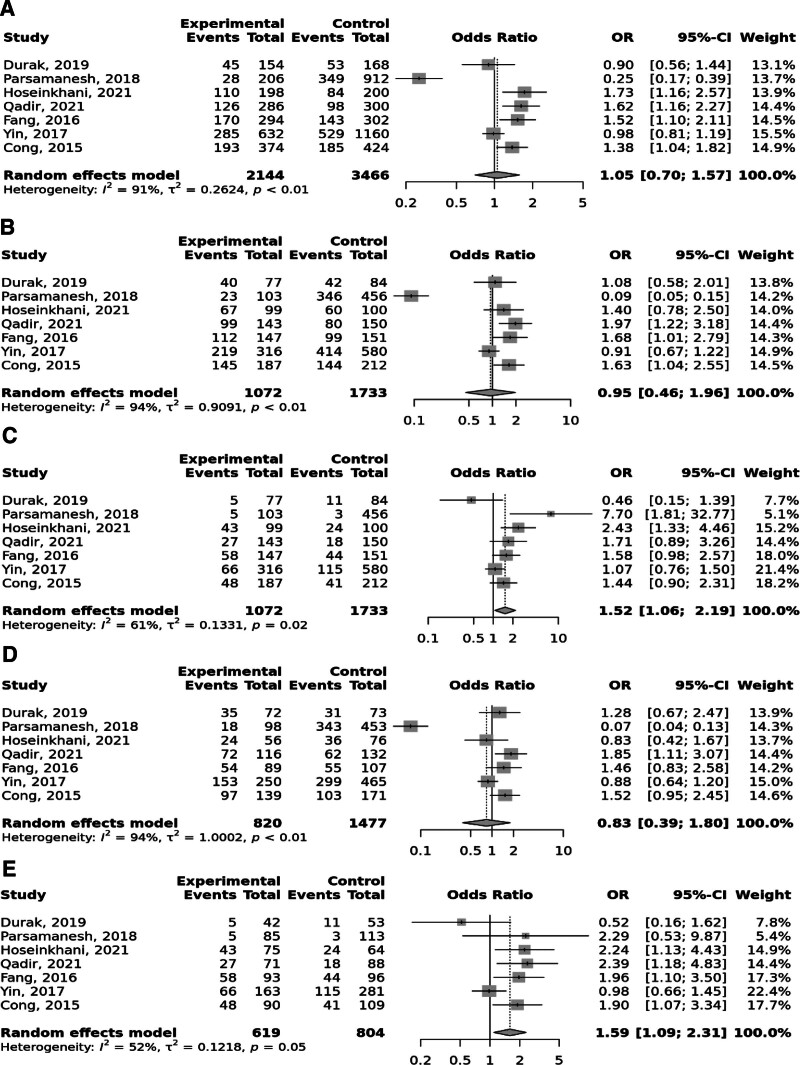
Meta-analysis of the association between FokI rs2228570 C > T polymorphism with gastric cancer in allelic (A), dominant (B), recessive (C), heterozygote (CT vs CC) (D), and homozygote (TT vs CC) (D) models.

**Figure 3.**
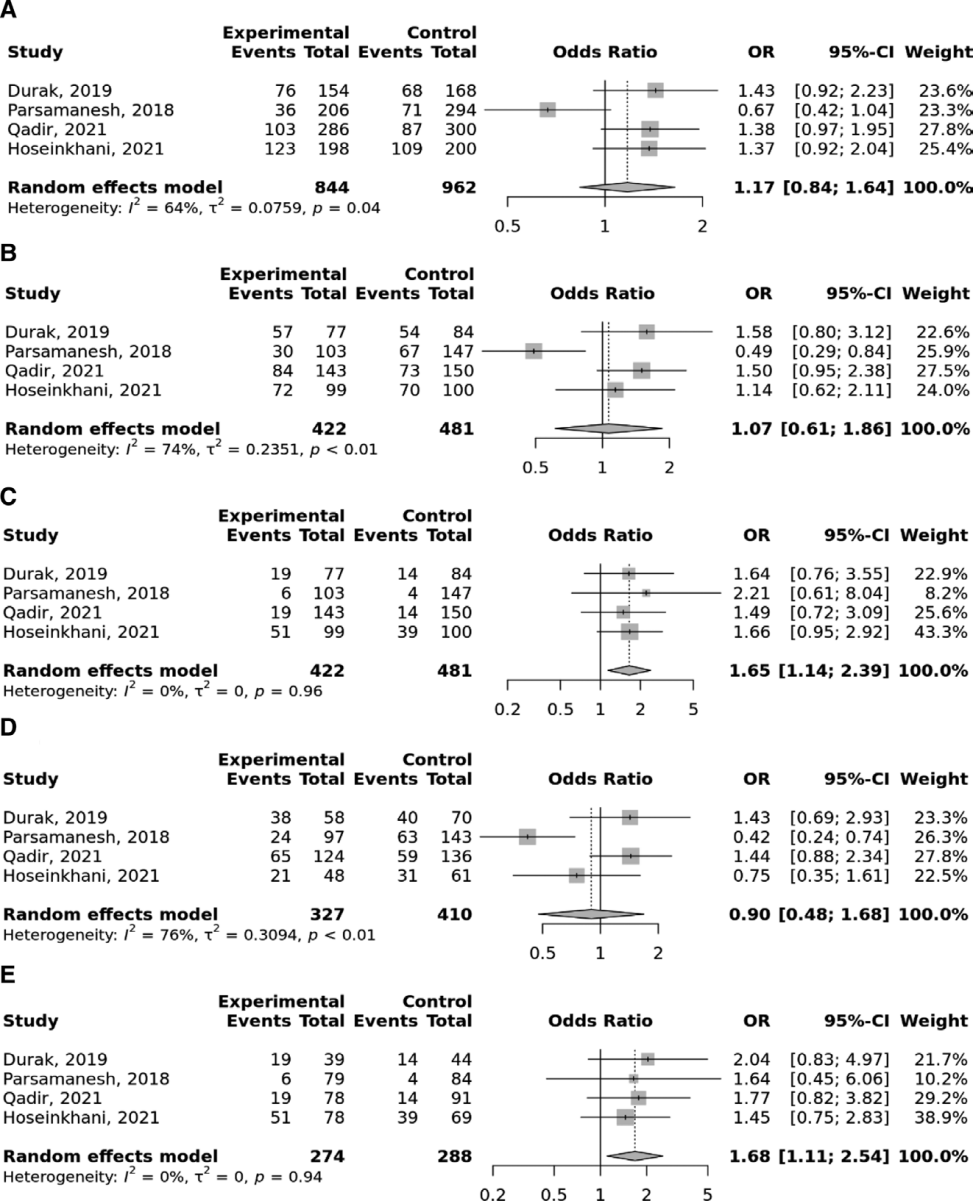
Meta-analysis of the association between Taq1 rs731236 T > C polymorphism with gastric cancer in allelic (A), dominant (B), recessive (C), heterozygote (CT vs TT) (D), and homozygote (CC vs TT) (D) models.

**Figure 4.**
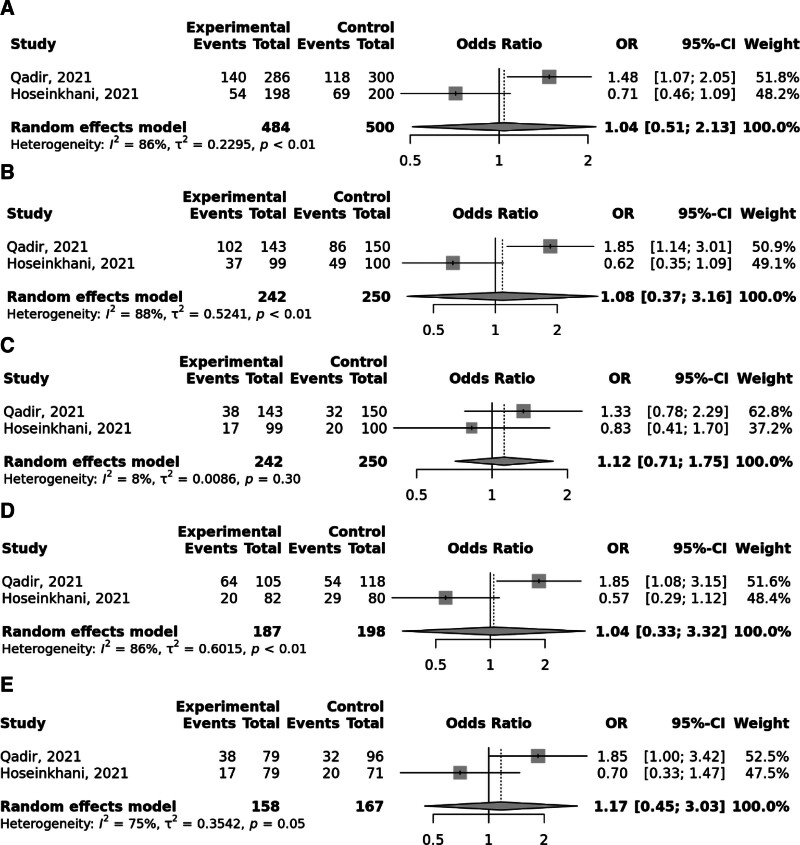
Meta-analysis of the association between BsmI rs1544410 G > A polymorphism with gastric cancer in allelic (A), dominant (B), recessive (C), heterozygote (AG vs GG) (D), and homozygote (AA vs GG) (D) models.

**Figure 5.**
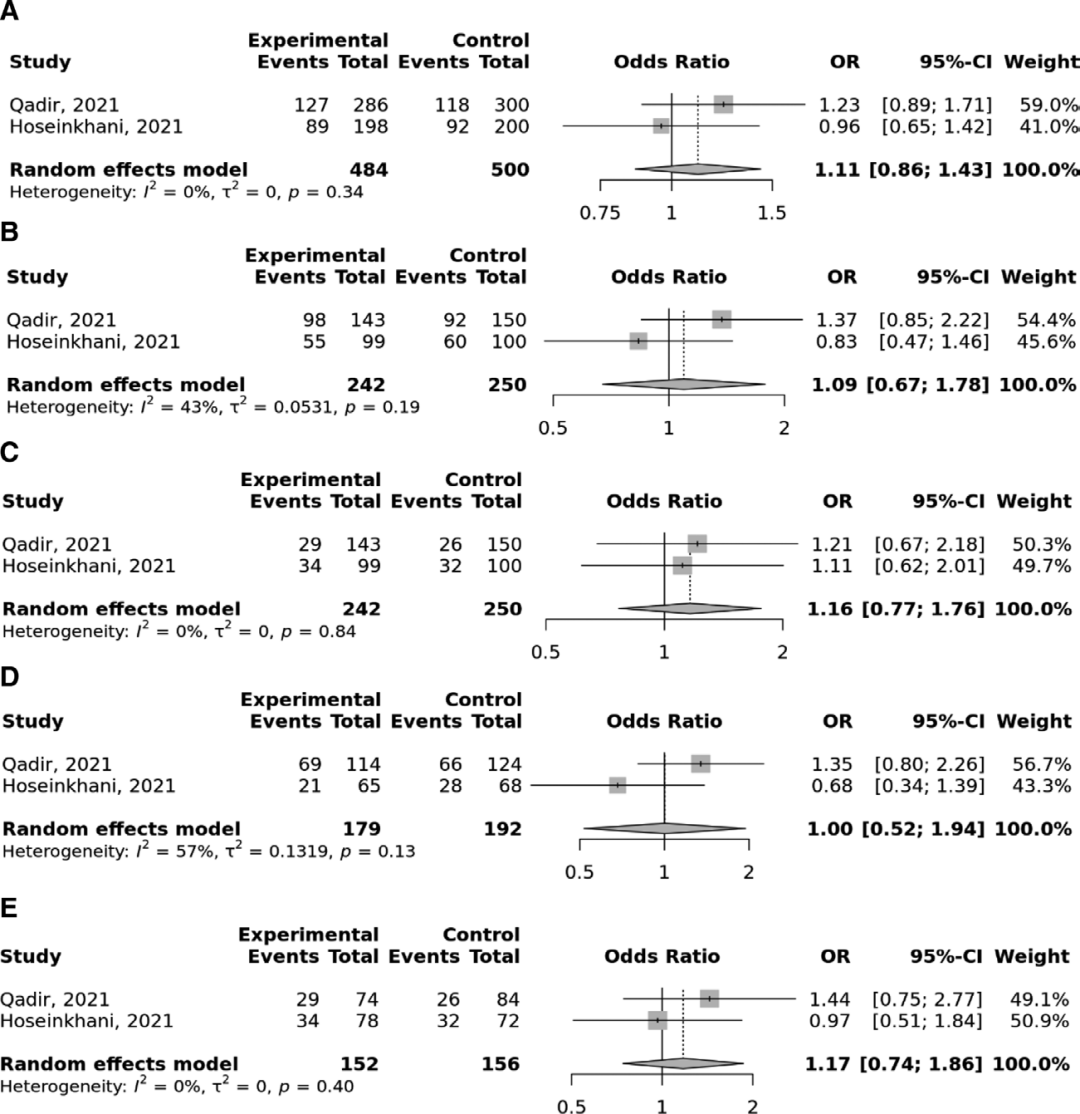
Meta-analysis of the association between ApaI rs7975232 G > T polymorphism with gastric cancer in allelic (A), dominant (B), recessive (C), heterozygote (GT vs GG) (D), and homozygote (TT vs GG) (D) models.

**Figure 6.**
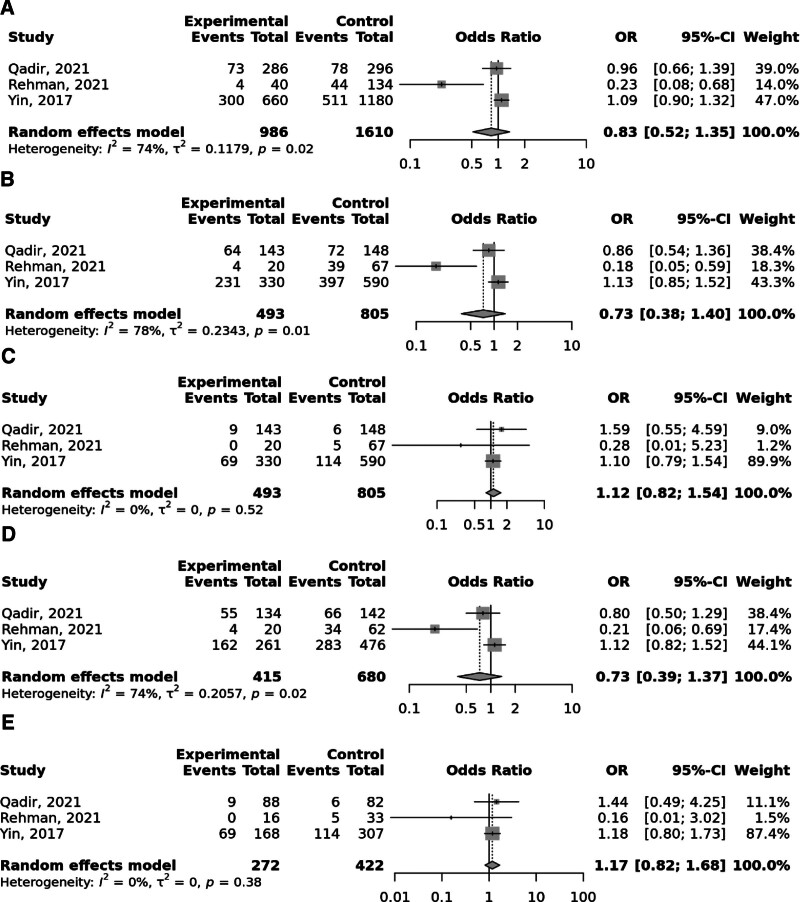
Meta-analysis of the association between Cdx2 rs11568820 G > A polymorphism with gastric cancer in allelic (A), dominant (B), recessive (C), heterozygote (AG vs GG) (D), and homozygote (AA vs GG) (D) models.

Overall, the FokI rs2228570 SNP was significantly linked to elevated odds to GC under the recessive and (OR = 1.52; 95% CI: 1.06–2.19, *P* heterogeneity = .02) and homozygote (TT vs CC; OR = 1.59; 95% CI: 1.09–2.31, *P* heterogeneity = .05) models, with significant heterogeneity among studies (Fig. 2). Moreover, Taq1 rs731236 was found to be linked to the elevated risk of GC in the recessive and (OR = 1.65; 95% CI: 1.14–2.39, *P* heterogeneity = .96) and homozygote (TT vs CC; OR = 1.68; 95% CI: 1.11–2.54, *P* heterogeneity = .94) models; no between study heterogeneity was observed (Fig. 3). No significant relationship was identified between BsmI rs1544410 (Fig. 4), ApaI rs7975232 (Fig. 5), and Cdx2 rs11568820 (Fig. 6).

### 3.3. Sensitivity analysis

In the sensitivity analysis, after excluding 3 studies which were not in HWE,^[12,21,23]^ the observed relationship between FokI rs2228570 polymorphism and GC was disappeared (recessive model: OR = 1.19, 95% CI: 0.83–1.72; homozygote model: OR = 1.33, 95% CI: 0.77–2.31), showing that this finding might be at risk of bias. For Taq1 rs731236, the pooled ORs remained consistent even after excluding the study by Hoseinkhani et al^[12]^ that did not adhere to HWE (recessive model: OR = 1.64, 95% CI: 1.004–2.68; homozygote model: OR = 1.84, 95% CI: 1.08–3.13), indicating the reliability of the findings.

### 3.4. Publication bias

**Figure 7.**
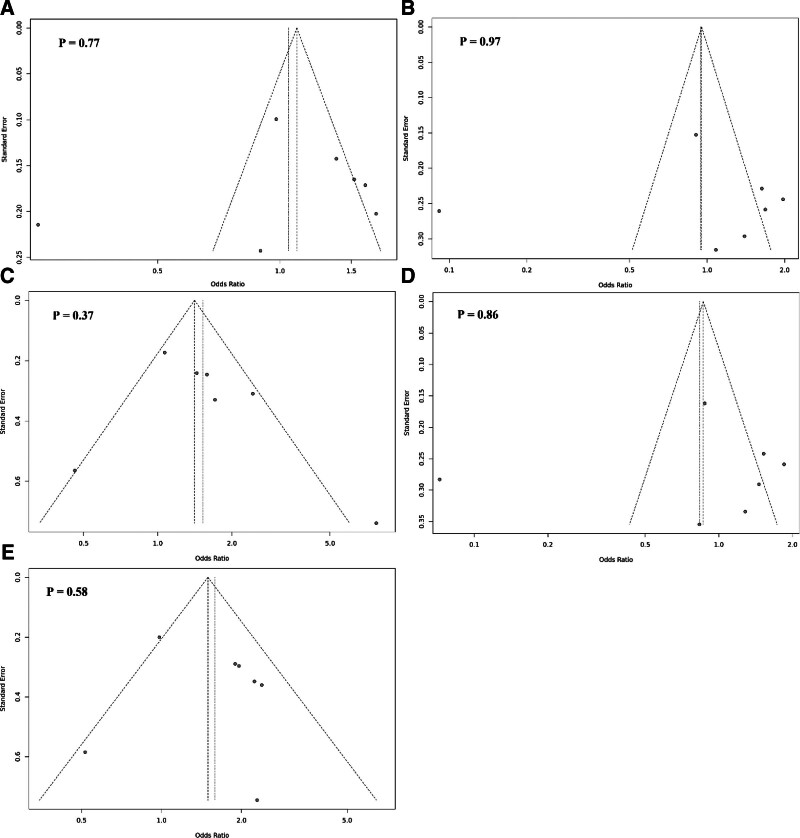
Funnel plot for publication bias in studies examining the association between FokI rs2228570 C > T polymorphism with gastric cancer in allelic (A), dominant (B), recessive (C), heterozygote (AG vs GG) (D), and homozygote (AA vs GG) (D) models.

The results of Egger test indicated the absence of publication bias for FokI rs2228570 polymorphism (Fig. 7). Due to the limited number of studies, publication bias tests were not carried out for other polymorphisms.

## 4. Discussion

This meta-analysis sought to investigate the association between common VDR variants and the odds of GC. The results indicated that Taq1 rs731236 polymorphisms was linked to a higher likelihood of GC. Furthermore, there was a weak evidence for the elevated odds of GC for the FokI rs2228570 SNP.

The relationship between VDR variants and GC has shown conflicting results in previous studies.^[7,23]^ In the study by Eom et al^[26]^ in a Korean population, VDR SNPs (rs4516035, rs1544410, and rs2239179) were not linked to GC. In contrast, in line with this meta-analysis, a case–control study on 564 Chinese subjects by Shen et al^[27]^ found a significant correlation between the TaqI polymorphism and GC and no association was found for ApaI. Variations in the findings of previous studies could be attributed to differences in sample size, ethnicity, or environmental factors like geographic location, dietary habits, and levels of physical activity. In this meta-analysis, a significant association of FokI and TaqI variants of VDR with GC was disclosed. Understanding these genetic variations could potentially aid in identifying individuals at higher risk for developing GC, allowing for targeted screening and early intervention strategies. Additionally, these findings may contribute to personalized treatment approaches and the development of novel therapeutic interventions tailored to individuals with specific VDR gene variants associated with GC susceptibility. The VDR gene polymorphisms examined in this meta-analysis, such as FokI rs2228570 and TaqI rs731236, are not specific to GC, as they have been associated with various other cancer types as well. The associations found are likely due to the broader role of the VDR gene in cancer biology, rather than being specific to GC. While these polymorphisms may influence the risk of GC, they are not necessarily specific to this cancer type. The VDR gene is involved in the regulation of various cellular processes, and its polymorphisms have been linked to the development of multiple cancer types. Further research is needed to identify potential GC-specific genetic markers or risk factors.

Multiple mechanisms mediated by VDR signaling contribute to its anticancer impacts. VDR signaling has the capability to prompt G0/G1 cell cycle arrest along with the increase in expression of various cell cycle inhibitors such as P21 and P27. Moreover, it can trigger cell cycle arrest at the G2/M phase and elevate the levels of E-cadherin and desmosomes, which promote apoptosis, improve adhesion, and inhibit the migration of cancer cells.^[12]^ The increased risk of GC associated with the FokI and TaqI SNPs may be justified by several biological mechanisms. Among VDR gene SNPs, the FokI variant is not in linkage disequilibrium with other polymorphic sites, indicating that it is independent in terms of genetic inheritance.^[28]^ A potential mechanism could involve alterations in VDR function/expression,^[29]^ leading to dysregulation of cell growth, differentiation, immune responses, inflammation, tumor suppression pathways, and apoptosis in gastric tissues.^[30–32]^ One key aspect of the FokI polymorphism is that it involves a T-to-C transition (ATG to ACG) in exon 2 of the VDR gene, where the ATG sequence encodes the translation-initiation codon of VDR mRNA (referred to as the T or f allele).^[17]^ This T-to-C transition leads to a change in the translation start site, causing the production of a truncated protein lacking part of the N-terminal activation domain.^[33]^ This alteration might affect the ability of the VDR protein to bind to its response elements or interact with cofactors, potentially impacting the regulation of downstream targets involved in cell growth, apoptosis, and inflammation – all processes relevant to carcinogenesis.^[12,17,29,34]^ Additionally, the FokI polymorphism has been linked to variations in serum vitamin D levels, which play a crucial role in maintaining normal cellular functions and preventing cancer progression.^[17]^ Lower serum vitamin D levels have been observed in populations carrying the FokI variant, suggesting that the polymorphism may contribute to impaired vitamin D signaling pathways.^[35]^

In contrast, the TaqI polymorphism in exon 9 also involves a T-to-C alteration, but in this case, it results in a synonymous change that impacts the mRNA levels of the VDR protein.^[7]^ TaqI polymorphism may modify alter transcriptional activity, and negatively affects VDR binding affinity to regulatory elements, which disrupt the balance between cell growth, differentiation, and apoptosis, thus promoting GC development.^[36,37]^ Although the TaqI polymorphism does not result in an amino acid change due to being synonymous, it may still indirectly affect the folding and conformational properties of the VDR protein, thereby influencing its interactions with ligands and corepressors.^[38]^ The T allele of the TaqI polymorphism is associated with increased transcriptional activity, mRNA stability, and elevated serum levels of 1,25(OH)2D3, the active form of vitamin D.^[38]^ Higher levels of 1,25(OH)2D3 may promote cell cycle arrest and inhibit cell proliferation.^[12]^ The TaqI polymorphism affects the binding affinity of VDR to specific regulatory elements, possibly leading to changes in gene expression patterns related to cell survival, proliferation, and apoptosis, which all are involved in carcinogenesis.^[12,39]^ Recent studies have suggested that VDR gene polymorphisms may influence the response to *H. pylori* treatment, which could have significant implications for the understanding the relation of VDR to GC.^[40]^ *H. pylori* infection is a well-established risk factor for the development of GC.^[41]^ The VDR gene plays a crucial role in the regulation of immune responses and inflammation, both of which are important in the context of *H. pylori* infection and gastric carcinogenesis.^[7]^ Some studies have reported that specific VDR gene polymorphisms, such as FokI rs2228570 and ApaI rs7975232, are associated with the success or failure of standard triple therapy for *H. pylori* eradication, with the CC genotype of FokI rs2228570 and ApaI rs7975232 SNPs being more resistant to eradication.^[40]^ These findings suggest that VDR gene polymorphisms may influence the host’s immune response and inflammatory processes, which in turn could affect the efficacy of *H. pylori* treatment. This could have significant implications for GC prevention, as the effectiveness of *H. pylori* eradication therapy might impact the risk of GC development. However, it should be noted that more extensive research is required to fully understand the complex interplay between the VDR polymorphism, vitamin D signaling, and GC development. Future investigations involving functional assays, mechanistic studies, and larger cohorts will help elucidate the precise roles of the VDR polymorphism in GC risk and provide valuable insights into potential therapeutic strategies.

To our knowledge, this is the first meta-analysis investigating the connection between VDR variants and the risk of GC. The strength of the study include a comprehensive overview of the association between 5 common SNPs in VDR and GC risk. Furthermore, we conducted a search for studies in all languages and incorporated research in Chinese, thereby reducing the likelihood of publication bias. Some limitations of this meta-analysis need to be taken into account when interpreting the findings. First, since studies recruited a combination of males and females and 8 out of 9 articles included in this meta-analysis were on Asian populations, subgroup analysis was not conducted to obtain possible gender- and ethnicity-specific associations. Thus, these findings may not be generalizable to other ethnicities, such as Africans and Caucasians. Future studies are required to explore the relation of VDR variants to GC in subgroups of males and females as well as in various ethnicities. Second, significant evidence of heterogeneity was observed for some of the SNPs. Third, the significance of gene-environment interactions and gene–gene interactions in the development of GC has been acknowledged. As a result of insufficient data from the primary studies, these interactions could not be taken into account in this meta-analysis. Lastly, in the sensitivity analysis, the relationship between the FokI rs2228570 polymorphism and GC disappeared after excluding studies that were not in HWE, suggesting that this finding may be susceptible to bias. Therefore, the pooled effect size for the FokI variant should be interpreted with caution, and it is important for this to be confirmed in future studies. Deviations from HWE can indicate issues with genotyping errors, population stratification, or other factors that may affect the validity of the genetic association findings. However, in certain populations, deviations from HWE may not necessarily indicate problems with the data, but rather reflect the complex demographic and genetic characteristics of the studied population. While several studies have identified significant links between genetic variations and diseases in cases where the genotype distribution in the control population departed from HWE, such deviations could potentially stem from genotype inaccuracies and biases in control selection.^[42]^ Therefore, in the sensitivity analysis, we excluded studies where genotype frequencies in the control population deviated from HWE to evaluate the stability of the combined effect estimates.

## 5. Conclusions

In conclusion, this meta-analysis disclosed that Taq1 rs731236 and FokI rs2228570 SNPs in VDR might be linked to GC risk. Addition research is required to validate this conclusion in different ethnicities. Future research should focus on exploring the interplay between VDR SNPs and environmental factors, thus helping to elucidate the relation of VDR SNPs to GC.

## Author contributions

**Conceptualization:** Min Guan, Yong Wang.

**Data curation:** Min Guan, Yong Wang.

**Formal analysis:** Yong Wang.

**Investigation:** Min Guan, Yong Wang.

**Methodology:** Min Guan, Yong Wang.

**Project administration:** Min Guan, Yong Wang.

**Supervision:** Yong Wang.

**Validation:** Min Guan, Yong Wang.

**Writing – original draft:** Min Guan, Yong Wang.

**Writing – review & editing:** Yong Wang.
